# Application of Multi-Omics Technologies to the Study of Phytochromes in Plants

**DOI:** 10.3390/antiox13010099

**Published:** 2024-01-14

**Authors:** Shumei Wu, Yue Gao, Qi Zhang, Fen Liu, Weiming Hu

**Affiliations:** 1 Basic Medical Experiment Center, School of Traditional Chinese Medicine, Jiangxi University of Chinese Medicine, Nanchang 330004, China; wushumei@jxutcm.edu.cn (S.W.); 20192029@jxutcm.edu.cn (Y.G.); 20171003@jxutcm.edu.cn (Q.Z.); 2 Lushan Botanical Garden, Jiangxi Province and Chinese Academy of Sciences, Jiujiang 332000, China

**Keywords:** phytochrome, multi-omics, photomorphogenesis, light signal, crop improvement

## Abstract

Phytochromes (phy) are distributed in various plant organs, and their physiological effects influence plant germination, flowering, fruiting, and senescence, as well as regulate morphogenesis throughout the plant life cycle. Reactive oxygen species (ROS) are a key regulatory factor in plant systemic responses to environmental stimuli, with an attractive regulatory relationship with phytochromes. With the development of high-throughput sequencing technology, omics techniques have become powerful tools, and researchers have used omics techniques to facilitate the big data revolution. For an in-depth analysis of phytochrome-mediated signaling pathways, integrated multi-omics (transcriptomics, proteomics, and metabolomics) approaches may provide the answer from a global perspective. This article comprehensively elaborates on applying multi-omics techniques in studying phytochromes. We describe the current research status and future directions on transcriptome-, proteome-, and metabolome-related network components mediated by phytochromes when cells are subjected to various stimulation. We emphasize the importance of multi-omics technologies in exploring the effects of phytochromes on cells and their molecular mechanisms. Additionally, we provide methods and ideas for future crop improvement.

## 1. Introduction

Light is an essential factor for the survival, reproduction, and development of all living things. Plants use photosynthesis to convert solar energy into chemical energy to meet their growth, development, and reproduction needs; thus, light is the foremost survival condition for plants [1]. Light is not only an energy source but also a signal. Plants use a complete set of photoreceptors to sense light signals and act on the external and internal environments of plants. By perceiving light signals in the external environment, spectral wavelength, light intensity, direction of light emission, and duration of day and night, photoreceptors allow plants to modify their morphology and physiology in a changeable environment to achieve the maximum capture and use of light [2]. According to the different wavelengths of light absorption, photoreceptors can be divided into phytochromes involved in the absorption of red light (R) and far-red light (FR) cryptochromes (CRYs) that play role in sensing blue light and ultraviolet light A (UV-A), phototropin (PHOT), aureochrome (AUREO), ZTL/FKF1/LKP2, and the ultraviolet B (UV-B) signaling receptor UV RESISTANCE LOCUS 8 (UVR8) [3,4,5,6,7]. This classification has two main advantages: (1) It ensures the fastest response. For example, it enables the precise resolution of light quality and other information through different light receptors, as well as regulates the development of chloroplasts and chlorophyll synthesis [8], and (2) it allows for specialization, such as when particular wavelengths trigger plants to bend and grow toward light to obtain the most suitable light. This phenomenon is ubiquitous in mosses, ferns, and angiosperms [9]. Blue and red lights enhance the phototropism of plants [10]. Photoreceptors also interact with each other to synergistically affect plant development [11].

A generic phytochrome is a water-soluble dimer protein comprising about 120~130 kDa proteins and a tetrapyrrole ring chromophore. Each monomer terminal folds into two main domains: N-terminal photosensory and C-terminal dimerization moieties [2]. Among them, the N-terminal domain is covalently linked to the tetrapyrrole chromophore that initiates a series of complex dynamics in response to light stimulation. The C-terminal domain promotes the dimerization of phytochrome and transmits light signals downstream [12]. At present, it has been determined that the model plant Arabidopsis (*Arabidopsis thaliana*) encodes at least five phytochrome members, namely phyA, phyB, phyC, phyD, and phyE, which have overlapping functions and can mobilize different responses depending on the source of light signals [13]. According to their stability, these phytochrome members can be divided into two categories: photo-unstable phyA, which principally exists in yellowing seedlings, decreases sharply under intense light irradiation and predominantly affects the seed germination and physiological response of plants in FR [14,15,16], whereas phyB, phyC, phyD and phyE are photostable and are mainly found in adult seedlings [17,18]. PhyB is the primary photoresponsive receptor, while other phytochromes act as secondary photoreceptors of phyB involved in regulating auxin synthesis, de-etiolation, seed germination, and flowering through various pathways [14,17,19,20].

Photoreceptors also exist in prokaryotes, but their structure and properties differ slightly from those in plants. The most remarkable chemical feature is that the homologous N-terminal structural domain of photochromes in prokaryotes binds bilins to produce photochromic holoproteins with a reversible photochromic effect [21,22]. Phytochromes of cyanobacteria exhibit phototaxis primarily by recognizing the quality and quantity of light. They are guided mainly by low-intensity green light, R, and FR, while high-intensity blue or UV-A light leads to negative phototaxis [23,24]. Many phytochrome family members are also found in fungi [25,26]. White collar 1 (Wco1) and phy1 are the main performers of fungal physiological functions under blue light and R/FR, respectively [27,28]. Contrary to phytochromes in plants, the two *Agrobacterium* phytochromes, Agp1 and Agp2, are mainly active in a dark environment to enhance the rate of plant infection [29].

**Figure 1:**
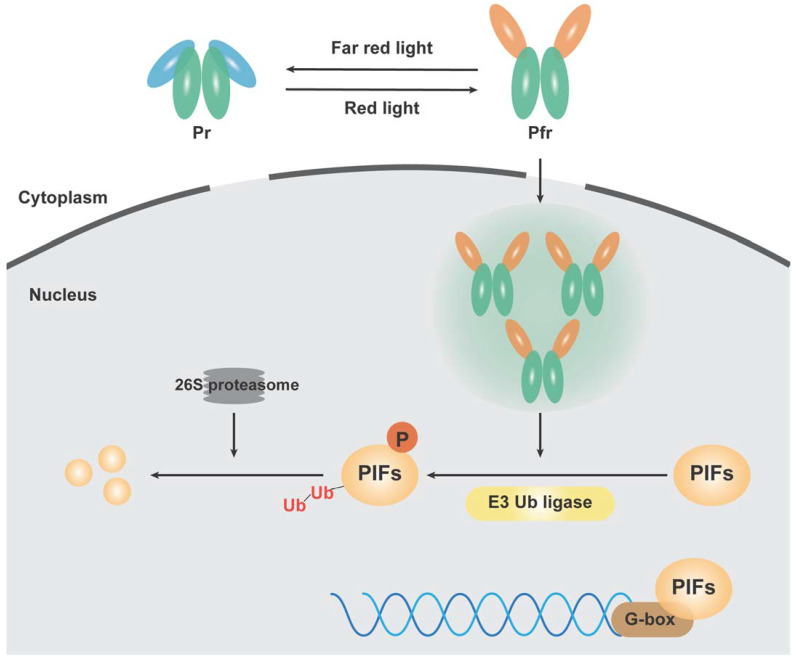
Schematic representation of phytochrome signal transduction mechanisms.

Phytochromes exist in two distinct photoreversible forms, the R absorption (Pr) and FR absorption (Pfr) isoforms, which can dynamically undergo reversible isomerization at the C15-C16 double bond [30]. The physiological activity of plants depends on the conversion ability between these two conformations [31]. After R irradiation, C15-Z anti-conformation is converted to C15-E. Inactive Pr isomerizes in the cytoplasm, is converted into active Pfr and then transferred into the nucleus via the nuclear localization activity of C-terminal [32], where it binds to the basic helix–loop–helix (bHLH) transcription factor subfamily, known as phytochrome-interacting factors (PIFs) [33], which all attach to the G-box (CACGTG) motif of light-regulated genes [34]. PIFs preferentially bind to the Pfr form of photosensitive pigments, leading to PIF sequestration, phosphorylation, polyubiquitylation, and 26S proteasome-mediated degradation [30,35,36]. This consequently induces the expression of target genes, various photomorphogenesis changes, and other physiological processes in plants (Figure 1). Arabidopsis has eight PIFs to maintain plant homeostasis [37,38]. Shang et al. demonstrated that PIF3 and PIF5 are positive regulators of the transcription and translation of abscisic acid (ABA) and other phytohormones, affecting growth by exposing seedlings to light for a long time in Arabidopsis [39]. PIF3 has also been found to co-promote chlorophyll and flavonoid synthesis with PIF1 [40], while PIF4/PIF5 and LONG-HYPOCOTYL5 (HY5) are antagonistically involved in plant temperature perception [41]. Pfr is rapidly degraded by protease under FR or shade and/or due to thermal mechanism and is reduced to inactive Pr, thereby reversing the related physiological response induced by Pfr [42,43]. Regulation of alternating growth and dormancy of plants between seasonal climate and diurnal light fluctuations is an evolved mechanism in adapting to complex environments. The phenomenon of phytochromes participating in plant biology is complex and changeable, and there are often many limitations in a single (omics) study. Analyzing and learning the changes in gene expression, protein production, and metabolites based on multi-omics technologies and perspectives can help explain the nature of light and facilitate the development of potential applications in plants. In recent years, multi-omics have been frequently applied to explain the physiological processes mediated by phytochromes (Table 1). This review evaluates the role of phytochromes from the perspectives of the transcriptome, proteome, and metabolome to provide a reference and ideas for future research.

## 2. Analysis of the Mechanism of Phytochrome Action Based on Whole-Genome Transcriptome

Plants immediately change the expression of transcription factors and downstream genes according to the changes in their surrounding environment. Transcriptomics studies investigate gene transcription and regulation rules in cells from an overall level, which can reveal genes involved in the whole process of plant light response and the subtle reactions that are not easy to observe. As a vital transcriptional regulatory factor, the role of phytochrome in regulating plant physiological functions has been studied using transcriptomics, which significantly promotes the elucidation of the regulatory mechanism of phytochrome.

The genes of higher organisms contain multiple selective promoters to expand their coding capacity. Changes in the selection pattern in response to stimuli often affect the translation efficiency or stability of mRNAs, and the encoded proteins change accordingly [74]. The diversity of proteome due to alternative promoters can ensure the maximum complexity of organisms. By changing the wavelength of light irradiation on wild-type (WT) and phyA and phyB double mutants, and conducting transcription start site sequencing (TSS-Seq) and mRNA-seq analysis, it has been demonstrated that phytochrome-dependent selective promoters change quickly. The light environment selects alternative promoters, which control transcription and selective splicing on the genome, and directly participates in whole-genome regulation and protein localization with plastid protein as the primary target [46], thus mediating the light response of plants. This reflects the adaptability of plants to their light environment, contributing to the higher biodiversity.

As sessile organisms, plants are mainly affected by unstable light and temperature, and in heterogeneous environments (high/low temperature, drought, etc.) with varying degrees of natural exposure to stress and resource competition, plants choose to respond to such stresses through morphological plasticity [75]. Based on bioinformatics analysis of microarray data, such as in the case of age-related and high-light (HL) stresses [76,77], the response of plants to heat stress (HS) is also through the hormone signaling pathway [41]. The apparent reaction of plants to HS in the natural environment is leaf senescence, and the removal of damaged tissues is selected to maintain the survival of young tissues, which is a strategy to improve the adaptability of plants to save resources under the condition of HS [78]. By comparing the Col-0 WT, phyA, and phyB mutants under HS, a previous study found that phyB is a molecular switch in HS response and is very sensitive to the HS signal [41]. High temperatures can control the expression and stability of PHOTOPERIODIC CONTROL OF HYPOCOTYL1 (PCH1) [79], reduce the activity of phyB, and protect the transcription of the carotenoid biosynthesis gene GERANYLGERANYL DIPHOSPHATE SYNTHASE 1 [44]. The downstream molecules of phyB, PIF4, PIF5, and HY5 are also affected [20,80,81], where PIF4 and its co-activator HEMERA (HMR) recruit a tail subunit called MED14 to activate auxin biosynthesis and signal transduction [82]. Another study found that slim shady is a mutant allele of phyB using the brassinosteroid–auxin–phytochrome (BAP) module [54] and a steroid phytohormone, brassinolide (BR), which promotes hypocotyl elongation [83], and was confirmed by RNA-seq analysis to be affected by phyB, which inhibits the transcription of BRI1-EMS-SUPPRESSOR 1 (BES1) gene and negatively regulates BR biosynthesis [53]. Finally, a plant thermal morphology with a shortened hypocotyl and reduced lateral root development is constructed, and these plants show higher heat resistance. PIF4 and PIF5 also have positive effects on leaf senescence under HL [77,80]. and three downstream molecules are also the target of phyB in inducing the leaf senescence morphology [52,84], with leaf senescence being a significant phenotype under HS. PhyB prevents leaf senescence by inhibiting the activation of PIF4 and PIF5 [84]. However, under FR, phyA interferes with the input of the phyB nucleus, inhibits the expression of genes related to chlorophyll (CHI) synthesis and age-related genes (SAG), and becomes a significant force in delaying leaf senescence [84,85]. The antagonistic effects between phyB and phyA on leaf senescence may induce the antagonistic regulation of the gene WRKY DNA-binding protein 6 (WRKY6) in the C3 and C4 clusters [52]. The presence of phyB appears to give plants a higher thermal tolerance, and further studies on phyB in breeding of heat-stress-resistant crops are needed. Early seed dormancy can preserve genes when plants are stressed [86], and improved breeding methods are also important. The state of seeds is regulated by phyB by altering the transcriptional REVEILLE1 mRNA and REV-EILLE1 levels to change the ratio of ABA to gibberellic acid (GA) [87]. Liao et al. used RNA-seq to identify a new signaling pathway, PHYB–WUSCHEL-RELATED HO-MEOBOX 11/12 (WOX11/12), which can regulate seed dormancy and hormones [50]. WOX11/12 was identified as a critical transcription factor in seed dormancy and germination, and a precise regulator of seed dormancy.

In addition, plants undergoing shade avoidance syndrome (SAS) immediately increase their photosynthesis and sacrifice their total biomass and number of leaves for early flowering to ensure successful reproduction with limited resources [88,89,90]. In Arabidopsis, it was found that phyB mutants lose their ability to interact with CONSTANS (CO) proteins and degrade them, resulting in early flowering [91], and this may also be related to the loss of interaction of HIGH EXPRESSION OF OSMOTICALLY RESPONSIVE GENES 1 (HOS1), a protein with ES ubiquitin ligase activity [92], which inhibits PIF4 function [93]. PhyA is antagonistic to phyB in controlling flowering [94]. More than 300 flowering-related genes were upregulated in the early stage of subterranean clover development, and a CCT motif related to CONSTANS and a FLOWERING LOCUS T (FT) b2-like protein was successfully identified under FR. These two genes, as well as their active downstream cascades, can induce flowering [95]. Interestingly, alfalfa upregulates the expression of legume homologs PIF3 and HB2 to mediate the occurrence of SAS, while it downregulates the expression of flowering induction factor SPL3 under a shaded environment [96]. Alfalfa undergoes chlorosis and delays flowering to store supplies, aiming to survive winter and reach the next breeding season. In contrast, long days (LDs) seem more beneficial to plants, and defense-related genes are greatly expressed in plants under LDs, especially resistance genes involved in jasmonate-dependent systems [55]. LD leads to phyB and phyC mutants of wheat having larger leaves and regulates the transcript levels of the flowering genes VERNALIZATION 1 (VRN1) and PHOTOPERIOD1 (PPD1), resulting in a delay in heading time compared to the short-day (SD) condition [56], which demonstrates that phytochrome is a key to ensuring the success of plant reproduction. Wang et al. found that targeting phyF regulates SELF-PRUNING 6A (StSP6A), florigen SELF-PRUNING 3D (SP3D) and StMADS1 to control flowering and tuberization of potatoes [58], while SD activates the overexpression of StSP6A, StSP3D and FLOWERING LOCUS of T-like 1 (StFTL1) to promote non-induced long-term tuber formation [97]. It is prospective to use the characteristics of phytochromes to improve crops. Controlling phyC in Foxtail millet can accelerate flowering, and C4 model plants obtained from phyC mutants, which could play a positive role in future research and development [57]. The phyC1C2 double mutant obtained through CRISPR/Cas9 recombinant technology prevented the occurrence of SAS, showing an improved variety more suitable for high-density cultivation [98]. Sun et al. overexpressed the downstream factor LONG HYPOCOTYL IN FAR-RED 1 (AtHFR1) of phyA to obtain a novel wheat variety resistant to osmotic stress and etiolation [99].

Photons are converted into potential energy during plants’ photosystem I (PSI) and photosystem II (PSII) reactions, and the process of energy flow is carried out by multiple thylakoid membrane proteins [100]. If unpredictable changes lead to the preferential excitation of a system or there is an imbalance between light and dark reactions, ROS will be gradually stored in plants. As the main site of photosynthesis, chloroplasts are rich in oxygen and have rich ROS sources, making them prone to oxidative damage [101]. Plastids are semi-autonomous organelles in plants, and tocopherols produced by plastids are crucial antioxidants to protect photosynthetic organelles [102]. Transcriptomics analysis of tomato fruits under a light environment showed that phytochrome regulated the degradation of PIF3, increased the expression of the GERANYLGERANYL DIPHOSPHATE REDUCTASE (SlGGDR) gene, and regulated the input of phytyl diphosphate (PDP) precursor, while tocopherol in fruits showed a phytochrome-dependent accumulation [49]. In a dark environment, the expression of tocopherol decreased in Arabidopsis [103]. There is a positive regulatory relationship between phytochrome and tocopherol. Viral infection can produce many ROS in plant cells, and the Receptor for Activated C-Kinase 1A (RACK1A) plays a role in this process [104]. Rice phyB mutants achieve drought tolerance by controlling their leaf area and stomatal density and mediating ROS clearance through ascorbate peroxidase and hydrogen peroxide (H_2_O_2_) enzyme [51]. The phyA and phyB1B2 mutants of tomato have a lower ROS content under drought conditions and can inhibit leaf film damage and maintain their water content to reduce oxidative damage [105,106]. Although heating, such as that during drought, depends on phyB activity mediating ROS accumulation, heating makes phyB mutants heat-resistant by depressing CO_2_ gas exchange and SPII activity [107]. Low concentrations of ROS serve as signaling molecules that trigger defense functions, but sustained stress can produce high concentrations of ROS, thereby reducing plant growth and yield [108]. PhyB may be a key link in plant clearance of ROS and maintaining the plant’s internal environment.

Due to environmental stress and evolutionary limitations, tomato and Arabidopsis are not identical in their phylogeny. The B-class phytochrome in tomato is subfunctionalized during gene replication to produce two homologs (phyB1 and phyB2) [109]. In previous research, it was revealed that the amino acids of phyB1 and phyB2 are similar to those of phyB and phyD in Arabidopsis [110], and the co-expression network of phyB1 and phyB2 mutants indicates that the fission phenomenon generated by subfunctionalization mainly occurs in auxin and light responses [48]. Arabidopsis grows phototropic via the combination of phyB and phyD [111], which is inhibited by phyB1 in tomato, while phyB2 is not associated with this reaction [48]. The effect of phyB and phyA on root geotropic growth of Arabidopsis is opposite to that of phyB1 [112]. Regarding photosynthesis, phyB1 exhibits apparent antagonism to phyB2 in its function to repress photosynthesis. In the process of subfunctionalization, phytochromes lose some tasks and may even exert antagonistic effects in the same process, while, at the same time, they retain synergistic and redundant functions [48]. For example, phyA, phyB1, and phyB2 have been described to play an essential role in the ripening process of fruits [44]. As a perennial tree, poplar alternates between growth and dormancy according to seasonal climate and contains only three phytochromes, including two phyB copies (phyB1 and phyB2) and phyA; among them, phyB1 and phyB2 have a strong sensing ability [113]. In Arabidopsis, phyB inhibits the activity of PIF4, PIF5, and PIF7 in response to photoperiod changes, while in poplar, whose response is also regulated by class B phytochromes, phyB only binds to PIF8, affecting the expression of FT and CENTRORADIALIS-LIKE 1 (CENL1) in response to seasonal growth [47]. It can be said that the subfunctionalization of phytochromes changes the physiological pathways of plants, which is an evolution that allows plants to balance their needs according to their environment.

Early studies showed that the unstable photosensitive pigment phyA mediated very low flux response (VLFR) and FR high-irradiance response (HIV) [114]. They were the only photoreceptors capable of receiving FR signals [115]. Increased protein levels of phyA in seedlings lead to densely grown plants, promoting petiole elongation and repositioning leaves to compete for sunlight resources to prevent the occurrence of SAS [116,117]. In a previous study using microarray analysis and GO analysis, phyA in tomato was found to be able to regulate the expression of intermediate (such as ABA INSENSITIVE3, ABI3) and SNF1-related kinase 1 (SnRK1) genes; subsequently inhibit respiration, amino acid synthase genes, and ATP production levels under a dark environment; and regulate carbon flux and energy supply distribution through glycolysis, β-oxidation, and the tricarboxylic acid (TCA) cycle to help plants conserve resources and maintain an energy-saving state under dark growth [45]. However, phyB and phyD dominate plant resource allocation under sunlight [70].

## 3. Application of Proteomics in the Analysis of Phytochrome

After sensing light, plants initiate a signal transduction series, and cells make appropriate physiological reactions to perform their functions. As essential substances, proteins directly participate in these reaction processes and are the tangible manifestation of life functions. Although proteomics cannot decipher gene expression and regulation mechanisms like transcriptomics, proteomics has significant advantages in analyzing the results of light perception and signal transduction. A comprehensive analysis of plant responses to ambient light based on different proteomic patterns or molecular patterns provides a powerful tool for understanding the overall molecular characteristics of cell responses to environmental stimuli.

The phyA Pr form does not play a physiological function in a dark environment, but phyA Pr transformed by phyA Pfr may have a physiological function [114]. However, Keisha et al. found that the upregulation of the TCA cycle in phyA mutants grown in a dark environment increased amino acid synthesis, with most of the carbon being allocated to protein reserves, and the plants had longer hypocotyls [45]. In general, plants stimulated by darkness utilize energy storage to achieve maximum axial elongation while limiting lateral growth and pigment production [118]. The WT uses malate synthase and isocitrate lyase to inhibit the TCA cycle, accelerate the breakdown of glucose and fat, and store carbon into carbohydrates, and has a shorter hypocotyl than the phyA mutant [45]. The hypocotyl length of Arabidopsis seedlings is negatively correlated with phyB activity and positively associated with phyA activity [119,120]. PhyA also plays a role in seedlings that have not been exposed to light.

When plants are transferred from darkness to expected light, root strengthening becomes the focus of plant development, closely related to sucrose transfer. PhyA can control sucrose distribution in seedlings by regulating sucrose transporter expression, promoting root growth while antagonizing other ways that inhibit root growth, and balancing the development of seedling roots and buds [45]. To better understand the mechanism underlying phyA function, Thomas et al. used isobaric tags for relative and absolute quantitation (iTRAQ) with high-throughput isobaric labeling to compare the protein difference between Ailsa Craig (AC) tomato seedlings grown under FR and those produced in the dark, and they found significant differences in proteins related to photosynthesis, carbon assimilation, and nitrogen metabolism [61]. Notably, the abundance of glycolate oxidase, which is involved in photorespiration and storage, and protochlorophyllide oxidoreductase B (POR B), which is a critical protein in chloroplast development, was significantly increased in the FR-treated group. In addition, the differences between phyA mutant seedlings and AC seedlings grown under FR were compared. It was found that the abundance of ATP synthesis-related proteins, glycolytic enzymes such as fructose-bisphosphate aldolase and phosphoglycerate kinase, proteins related to nitrogen metabolism, Calvin cycle enzymes such as sedoheptulose-1,7-bisphosphatase (SBPASE), proteins related to carbon assimilation and carbohydrate metabolism all decreased; these proteins target chloroplasts [61]. PhyA-regulated genes can be divided into ‘early response’ genes with low expression flux and ‘late response’ genes with high expression flux according to their time course of term [16]. In a previous study, the accumulation of differential proteins occurred after 48 h, and the maximum abundance was observed after 96 h [61]. It can be said that FR activates plant photosynthesis and chloroplast development. The absence of phyA leads to an overall downregulation of the abundance of photosynthetic and chloroplast-related proteins [61], and phyA likely establishes a de-yellowing function through late reaction. Interestingly, a portion of the chlorophyll AB-binding protein (CAB) in the chloroplast light-collection complex of WT Arabidopsis was found to be highly upregulated under R, while there was little change under FR, possibly due to FR blocking in the longer FR greening response [63]. PhyA appears essential in guiding photosynthesis under FR and has potential applications in preventing the unnecessary elongation of densely grown plants.

The moon also reflects low-intensity sunlight, although not enough to be used by plants, researchers are still exploring lunar agriculture [121,122]. It was confirmed in a recent study that moonlight affects plants’ life cycle, and the moon’s state at the time of planting is related to germination, growth, and flowering of seeds [122]. Increased levels of phyB and Phot2 proteins in mustard plants exposed to the full moon were accompanied by increased levels of stress-related proteins and ROS detoxification enzymes, resulting in accelerated growth of mustard plants [67]. Thus, moonlight may also be an essential light signal for plants.

As a SAS repressor, phyB controls various plant functions through reciprocal signal regulation with phyA under R [112,123]. In Arabidopsis *phyAphyB* double mutants, declines in the abundance of chloroplast and mitochondrial target differential proteins have been identified based on two-dimensional gel electrophoresis (2-DE). This includes different types of metabolic enzymes, including proteins involved in stress and defense, proteins with binding function or cofactor requirements, storage proteins, energy proteins, protein-fate proteins, and featureless functional proteins, in particular, the critical photorespiratory protein, glycine decarboxylase P-protein, has a profound impact [62]. Interestingly, the abundance of the α subunit of heterotrimeric G-protein (Gα) is upregulated, and the inactivation of phyB and phyA enhances plant response to light. Since other phytochrome lose their phyA antagonism, the double mutant upregulates the Gβ protein under R, which inhibits hypocotyl elongation and downregulates enzymes related to cell wall synthesis and carbon metabolism to shorten the hypocotyl [62].

To improve the efficiency of photosynthesis during variable quality and quantity of daily sunlight, the endogenous biological process that occurs with an oscillation of about 24 h, which is termed as the circadian rhythm and also known as the circadian clock, is used to detect changes in sunlight/darkness and temperature in the daily environment. It also regulates the balance between the PSI and PSII systems and the homeostasis of ROS [124]. A study based on thylakoid proteomics has proved that plants use photosystem stoichiometry to regulate the imbalance of light and electron transport. This domestication reaction is a photochrome-mediated process that occurs mainly through chloroplast regulation of PSI to adjust the relative abundance of the two photosystems, which is particularly closely related to phyB [66]. Warm temperatures can inactivate phyB that chloroplasts and affect the photosystems [44].

To discover the connection of phytochromes with plant ROS metabolism, Marketa et al. captured the night–day variation in the protein spectra of four mutants of phytochromes (phyA, phyB, phyC, and phyD) and one mutant of the circadian clock gene (*lhy*, LATE ELONGATED HYPOCOTYL), and about 640 proteins showed differences; most of the identified pathways were enriched in proteins related to protein synthesis, photosynthesis, redox metabolism, amino acid biosynthesis and biosynthesis of secondary metabolites [65]. Leaf movement, flowering time, and CO_2_ assimilation were also affected, and these differences were more significant in phyB mutants [65]. More than 300 novel oscillations were successfully identified from these differentially expressed proteins [65]. Oxidoreductase can help cells clear ROS in vivo, and its level rises to a peak at noon [125]. The study pointed out that the hydrogen peroxide content of the four phytochrome mutants decreased. However, oxidoreductase was still enriched in the four mutants, and the night–day variation in hydrogen peroxide in the phyD mutants did not differ and offered little specific information [65]. PhyD may scavenge ROS metabolism through the glutathione metabolic pathway. Phytohormones are regulated by the biological clock [126], and the level of cytokinin-reactive protein is strongly positively correlated with the expression of phyB and phyD in the dark [65]. It can be postulated that, as they are controlled by the biological clock [127], phytochromes affect the circadian rhythm of plants, and the synthesis of phytohormones is the pivotal link in the antioxidant system of plants. This theory supports the role of phytochromes in optimizing plant growth performance, particularly with current climate change.

After receiving light, phytochromes transmit signals to cells through protein–protein interactions. Identifying proteins that directly or indirectly interact with phytochromes is crucial to understanding plants’ light signal transduction pathways. For example, PIF3, nucleoside diphosphate kinase-2 (NDPK2) and phytochrome kinase substrate 1 (PKS1) proteins are all known to interact with phyA and phyB [128,129,130], while AR-RED ELONGATED HYPOCOTYL1 (FHY1) and its homolog Fhy1-like (FHL) interact directly with phyA for photo regulation [116]. Co-immunoprecipitation (Co-IP), followed by proteomic analysis, is an effective means to screen for phytochrome-interacting proteins. Bong-Kwan et al. identified seven proteins interacting with phyA and nine proteins interacting with phyB, and the protein phosphatase type 2C (PP2C) and 66 kDa protein mutants exhibited a phenotype consistent with the phenotype of phyB mutants [64]. The PP2C and 66 kDa proteins were proposed to be new phyB-interacting proteins, and the 66 kDa proteins were identified as light-signaling components in early plant development.

## 4. Metabolomic Analysis to Assess the Effects of Phytochromes on Plants

Metabolomics is a new omics technology that studies the differences and metabolic mechanisms of biological metabolites using nuclear magnetic resonance (NMR) and mass spectrometry (MS), and allows for a comprehensive qualitative and quantitative analysis of metabolites [131]. Compared with other omics technologies, metabolomics can more directly reflect the information of organisms. As critical regulatory factors in plant biomass, carbon supply, and metabolism, phytochromes participate in multiple metabolic pathways. Therefore, studying the regulation effects of phytochromes on plant metabolites and analyzing the complex metabolic network of plants has far-reaching significance for plant growth and management.

Photosynthesis is a process in which plants use phytochromes to sense light and use light energy to fix carbon from CO_2_ and release molecular oxygen [132]. However, in the process of growth and development, plants may encounter darkness or shading stress, which inactivates phyB in the plant canopy [133] and prevents the germination of seeds on the soil surface [134]. In contrast, phyB mutants can still germinate [135]. Leaves turn yellow due to plant chlorophyll degradation [136,137], a prominent feature of plant aging [138]. Plants that develop SAS mainly using sucrose produced by the decomposition of neutral lipid triacylglycerol stored in seedling as the primary energy source before germination [139]. The plant growth rate slows down while the stem, petiole, and hypocotyl begin to extend [140,141], sacrificing the development of cotyledon and roots to break through the barrier and increasing the risk of lodging. In previous metabolomics analysis, primary and secondary metabolites, such as niacin, alkaloids, phenylpropanoids, glucosinolates (GSLs), and flavonoids, were affected [72]. If light is restored, phytochromes regulate carbon flux through the primary metabolic pathways [37]. In addition, they upregulate glycometabolic enzymes and thylakoid synthetase-related genes, significantly decrease soluble sugar (including glucose and fructose) and starch levels, and enhance the regulation of oil body mobilization [72], thus revealing a crucial role of photochromes in coordinating the metabolism of sugar and oil in plant de-etiolation. When chlorophyll conversion is accelerated, the chloroplast content [72],the Calvin cycle, biosynthesis of chlorophylls, carotenoids, isoprenoid quinones, thylakoid lipids, sterols, and amino acids are notably increased during de-etiolation [68]. Beyond that, phytochromes also coordinate HY5 and PIF to promote cell wall and chlorophyll synthesis [40].

PhyA and phyB are two very critical photoreceptors in plants. PhyA has a dramatic effect on several primary metabolites in Arabidopsis, including amino acids, starch, organic acids, and sugars under R and white light [142], which was also confirmed in metabolomics experiments of tomato phyA mutant and WT tomato seedlings. The carbohydrate and TCA cycle intermediate levels increased in tomato mutant seedlings, and the amino acid levels were more remarkable [61]. PhyB played an essential role in regulating starch storage only when plants were exposed to low light or R: FR (11.8 ± 0.6) and during the photostationary state of photochrome (0.855 ± 0.001) [69]. In a long-term study, Xiaonet al. found that the photosynthesis of the phyAB double mutant of Arabidopsis was not affected compared with the WT. Still, the number of plastoglobuli was significantly reduced [69]. Plastoglobulus, found in the lipoprotein granules of chloroplasts, is a type of plastid microsphere that increases during the oxidative stress and senescence of plants [143]. In addition, the erythritol and galactose involved in carbohydrate metabolism, glyoxylate, and TCA intermediates, as well as ethanolamine and octadecanoate in the lipid metabolism pathway, aspartic acid, and homoserine, were significantly decreased in the mutants. The overall biomass did not change, but starch accumulation was notably reduced [69]. The possible cause is the deletion of phyAB, which downregulates the downstream glucose-1-phosphate adenylyltransferase small subunit (APS1) and NDPK2, thereby affecting starch synthetase [69]. The control of plant primary metabolism by photochromes described by Xiao et al. [69] supports the findings of the study on phyBD, phyABD, and phyABDE mutants by Deyue et al. [70]. The biomass of the mutant plants decreased significantly, and the fixed CO_2_ decreased while carbon allocation changed. Metabolites such as threonine and succinic acid, whose levels were positively correlated with the starch content, increased significantly in the mutants at dusk. The levels of starch and sucrose contained in the mutants matched those of the WT or were even higher, and sugar metabolism was active, especially in the plant stems and roots. The growth rate reached the level of the WT until the end of the night, while the WT had a higher biomass and a faster overall growth rate [70]. Phytochrome changes plants’ night/day growth ratio, which is required for plant growth. In addition, the mutants accumulated TCA intermediates, amino acids, sugar derivatives, and stress metabolites such as proline and raffinose [70], reducing their sensitivity to ABA and salt and enhancing plant resistance, which seems to be a strategy for plants to survive with limited resources [144,145]. Other scholars found that plant biomass significantly increased in low-temperature environments, and plant response to low R: FR was regulated by temperature [140]. At 16 °C, glycine, which can enhance plant resistance, is significantly increased in plants growing at low R: FR [141], and the receptor-like kinase (ERECTA), which affects leaf area prolapse [146], contributes to petiole elongation [140], allowing plants to obtain maximum light energy while reducing freezing damage.

In rice, only the phyA, phyB, and phyC phytochrome families affected rice yield, quality, and grain characteristics [147]. A rice phyB-deficient mutant was obtained via the CRISPR/Cas9 technique. Compared with the WT, the starch complex of the phyB mutant swelled rapidly in the seed through the lipid metabolism pathway; organic acids, sugars, amino acids, phytohormones, and lipids were all deficient, and carbon in the seed was re-distributed [73]. Grain size and chalkiness content are meaningful indicators of rice seed quality [148,149]. PhyB profoundly affects rice yield and quality, and further study on the mechanism of photosensitive pigments will be helpful for future rice breeding. Besides improving breeding, increasing the seed germination rate is also essential for plant development. PhyB mainly activates seeds for germination, and exposure to R can start and enhance the seed germination rate [150]. Puthanvila et al. [71] proposed using a He-Ne laser as a biological stimulator of seeds. After laser irradiation, plant primary metabolites were markedly increased, and phyA expression was upregulated. With an increase in the content of GA combined with GA antagonists, the ABA content decreased, seed dormancy was broken, and the germination rate was higher. In addition, using a He-Ne laser to regulate plant secondary metabolites during the seedling period significantly enhanced the plant’s photosynthetic and metabolic rates [71]. Another study found that using a He-Ne laser had the same effect on the seeds of sage and could accelerate the expression of the phyB gene and mediate the production of H_2_O_2_ to improve the influence of the saline–alkali environment on seedlings [151]. These results verified the effectiveness of He-Ne lasers in plant cultivation. In addition, the environmental safety and convenience of He-Ne lasers may provide an essential means of crop improvement in the future.

## 5. Application of Omics to Analyze Epigenetic Changes Associated with Phytochromes

Undoubtedly, changes in light/dark conversion and temperature are often involved in changes in plants’ internal gene expression. Willige et al. and Kim et al. found shadow changes the chromatin remodeling [152,153]. To explore the role of epigenetic factors, Calderon et al. conducted a comprehensive analysis using RNA-seq and ChIP-seq. They found that histone three lysine four trimethylation (H3K4me3) plays a crucial role in this process [59]. Shadow stimulation triggered phytochrome target gene expression mediated by PIF, an upregulation of the H3K4me3 level occurred after the change in the target genes, and the speed was slow. H3K4me3 stabilizes the transcription of target genes to buffer the conversion of light and dark [59].

It is interesting to investigate the epigenetic effects of phytochromes on the regulators of fruit ripening in tomato. During fruit ripening, phytochromes perceive signals from light and temperature, and affect chromatin organization factors and transcriptional regulators, such as DNA methylase/demethylase, chromatin-remodeling factors, histone modification enzymes, and ripening-associated transcription factors, thus regulating tomato fruit ripening through a precise gene expression network. In tomato, phyB1B2 has a more significant effect on fruit ripening than phyA, although both phyA and phyB1B2 affect the global methylome, transcriptome and sRNAome [60]. Epigenomic reprogramming can be regarded as a molecular switch for fruit ripening by coordinating phytochromes to control fruit traits.

## 6. Conclusions and Future Perspectives

**Figure 2:**
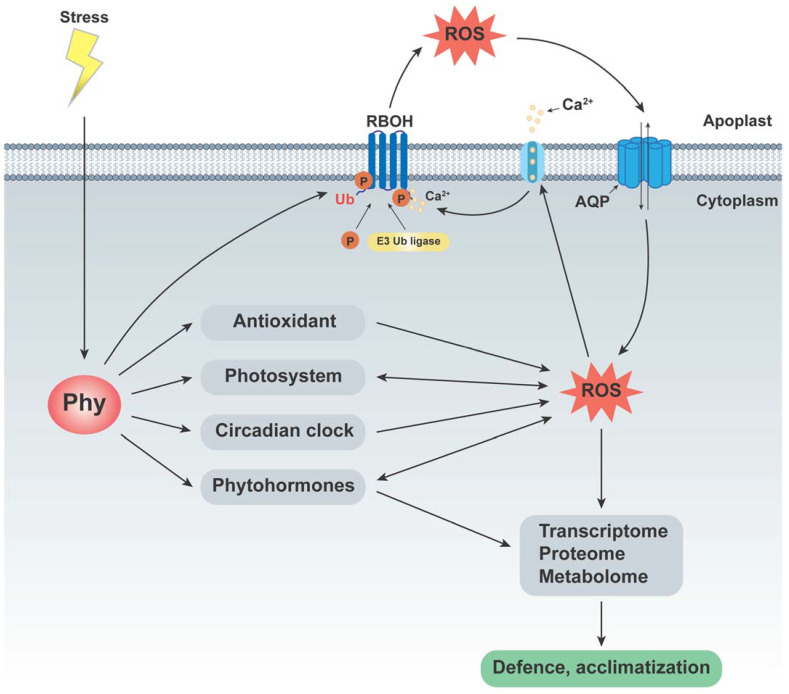
Phytochromes play a crucial role in ROS-mediated stress responses.

ROS are by-products of cellular metabolism and, when in excess, induce oxidative stress and oxidize many small molecules in plant cells [154]. A sophisticated system exists to support normal plant metabolism and keep the dynamic balance between ROS production and scavenging [155]. The tight regulatory link between the ROS system and phytochromes has gradually been uncovered using omics technology. Phytochromes function as light-signaling responders to transmit environmental information received from the sensor to the respiratory burst oxidase homolog (RBOH) protein, which triggers the production of ROS in the apoplast and organelles through Ca^2+^ signaling, phosphorylation, and ubiquitination reactions [156,157,158,159]. ROS can diffuse freely through the plasma membrane (PM)-localized aquaporin (AQP) and are translocated to different compartments, thereby triggering distinct signaling pathways depending on the stress encountered [160,161] and activating stress-specific adaptation and defense mechanisms; one of the most effective stress responses depends mainly on the action of phytohormones. Neighboring ROS can activate Ca^2+^ channels [162], and these signals collectively reprogram the transcriptome. To preserve ROS balance, phytochromes inhibit the AQP within them [105] and trigger antioxidants, photosystems, the circadian clock, and phytohormones [49,65,66,160,163]. Phytochromes link ROS signals to phytohormones and other stress response signaling pathways, thereby regulating plant growth and death [160], defense responses [104,161], stress tolerance [51,107], senescence [69], etc. (Figure 2). In particular, they have been extensively studied for their ability to enhance plant adaptation to stress, and this improved resistance can be sustained or passed on to the next generation via ROS-related epigenetic mechanisms [164,165,166,167].

Multi-omics techniques have constituted a widely applied research methodology in recent years, particularly the analysis of transcriptomics, proteomics, and metabolomics (Table 1). By investigating the close connection between cell biological mechanisms and ecological theory, the mutual verification of different omics techniques can successfully explore organism changes at the microscopic level and discover deep-level life activities. The use of omics techniques has become a ‘Rosetta stone’ in the field of botany, offering research results that are forward-looking and targeted. Existing studies have greatly enriched the knowledge of phytochromes’ regulation of light-response mechanisms. However, the precision of these techniques is still not enough; the function of phytochromes in bacteria and fungi is poorly understood, and more research is required to fully understand the crucial regulatory role that phytochromes play in the ROS system, which is essential to understand the organisms’ responses to environmental stimuli. It is necessary to continue to expand omics technologies and strengthen the application of multi-omics technologies to study phytochromes to promote modern agriculture.

## Figures and Tables

**Table 1:** Omics studies on phytochromes.

Transcriptomics				
Species/Tissue	Experimental Condition	Platform	Key Points of Interest	Ref.
*A. thaliana* seedling	Heat stress treatment	Microarray	Phytochrome (phy) B is an essential thermal sensor in plants.	[41]
Tomato fruit	Normal growth conditions	RNA-seq	The effect of phyb1b2 on tomato fruit ripening is greater than that of phyA.	[44]
Tomato seedling	Seedlings grown in the dark and exposed to red light (R) treatment	RNA-seq	PhyA helps tomato seedlings maintain growth in the dark.	[45]
*A. thaliana* leaf	High R: far-red light (FR) and low R: FR	RNA-seq	The phytochrome-dependent selective promoter is involved in genome-wide regulation with changes in light wavelength.	[46]
Hybrid aspen Populus *tremula × tremuloides* bud	After 12 weeks of exposure to 14 h light/10 h dark, the temperature dropped to 4 °C for eight weeks, and then plants were exposed to 21 °C and 18 h light/6 h dark	RNA-seq	PhyB in poplar regulates the seasonal growth of trees.	[47]
Tomato seedling	Dark environment and fast harvest under green light and rapid R	RNA-seq	The subfunctionalization of phyB is mainly related to auxin and photosynthetic responses.	[48]
Tomato fruit	Normal growth conditions	RNA-seq	The activation of PIF3 by phytochrome increases the content of tocopherols in fruits.	[49]
*A. thaliana* seed	Normal growth conditions	RNA-seq	A new phyB downstream transcription factor, WUSCHEL-RELATED HO-MEOBOX 11/12 (WOX11/12), was identified as a precise regulator of seed dormancy.	[50]
Rice seedling	Drought stress	RNA-seq	Twenty-nine drought-tolerance genes were identified, and the scavenging effect of phyB on reactive oxygen species (ROS) in rice was demonstrated.	[51]
*A. thaliana* leaf	R or FR	Microarray	There are antagonistic effects of phyA and phyB on leaf senescence.	[52]
*A. thaliana* seedling	R	RNA-seq	PhyB negatively regulates the BR signaling pathway.	[53]
*A. thaliana* seedling	indole-3-acetic acid (IAA) treatment	RNA-seq	Slim shady is a mutant allele of phyB.	[54]
*A. thaliana* leaf	Short days (SD) or long days (LD)	Microarray	Long days improve plant resistance.	[55]
*Triticum turgidum* L. leaf	SD or LD	RNA-seq	PhyB and phyC are essential for wheat flowering.	[56]
*Setaria italica* seedling	Normal growth conditions	RNA-seq	C4 model plants were successfully developed.	[57]
*Solanum tuberosum* L. leaf	Normal growth conditions	RNA-seq	PhyF has regulatory effects on flowering and stem fragmentation in potato.	[58]
**Epigenetics**				
**Species/tissue**	**Experimental condition**	**Platform**	**Key points of interest**	**Ref.**
*A. thaliana* seedling	Simulated shade (30 mmol m^−2^ s^−1^, R/FR ~ 0.3)	RNA-seq and ChIP-seq	Histone 3 lysine 4 trimethylation (H3K4me3) buffers light fluctuations in plants.	[59]
Tomato fruit	Normal growth conditions	MethylC-seq, RNA-seq, and sRNAome	The changes in the mRNA profile of maturation-related genes induced by phyB1B2 involve DNA methylase/demethylase, histone-modifying enzymes, remodeling factors, and transcriptional regulatory factors.	[60]
**Proteomics**				
**Species/tissue**	**Experimental condition**	**Platform**	**Key points of interest**	**Ref.**
Tomato seedling	In the dark or under continuous FR	iTRAQ	PhyA is the primary regulator of tomato under FR.	[61]
*A. thaliana* seedling	Culture under 4 °C in darkness for 4 days, and then transfer to continuous R at 23–25 °C for growth	MALDI-TOF-TOF MS	Double mutants under R shorten their hypocotyl and regulate proteins involved in stress and defense, proteins with binding function or cofactor requirements, storage proteins, energy proteins, protein-fate proteins, and featureless functional proteins, especially essential photorespiratory proteins.	[62]
*A. thaliana* leaf	R, FR, and blue light	MALDI-TOF MS	PhyB mutants retain physiological responses to R, but phyA mutants do not respond to FR, and the wild type under FR has little effect on chloroplast-associated proteins.	[63]
*A. thaliana* seedling	After four days of dark growth, apply R for 20 min	MALDI-TOF MS	Protein phosphatase type 2C (PP2C) and a 66 kDa protein are new proteins discovered to interact with phyB.	[64]
*A. thaliana* seedling	12 h photoperiod, with harvest in the middle of the light or dark period	RPLC-MS	Three hundred novel oscillations were identified in the four mutants, and most of the proteins were enriched in the ROS metabolic pathway.	[65]
*A. thaliana* leaf	Plant’s photosystem I (PSI) light or plant’s photosystem II (PSII) light, with 16 h light/8 h dark or 8 h light/16 h dark	LC-MS/MS	The light quality response of PSI gene transcripts and proteins is closely related to phyB.	[66]
Tomato seedling	Dark environment, with fast harvest under green, safe light, and rapid R	MALDI-TOF-TOF MS	PhyA enhances the enzymatic upregulation of glycolysis, β-oxidation, and the tricarboxylic acid (TCA) cycle that accelerates the breakdown of glucose and fat. It also increases the abundance of storage proteins and controls the distribution of sucrose in seedlings by regulating the expression of sucrose transporter to ensure the development and growth of seedlings.	[45]
*A. thaliana* leaf	Continuous R after 4 days of dark growth	MALDI-TOF-TOF MS	The abundance of the α subunit of heterotrimer g protein (Gα) in phyAB double mutants is upregulated, and sensitivity to light is enhanced.	[62]
*Nicotiana tabacum* seedling	full moonlight	GC-MS	Moonlight is also a critical light signal for plants.	[67]
**Metabolomics**				
**Species/tissue**	**Experimental condition**	**Platform**	**Key points of interest**	**Ref.**
*A. thaliana* seedling	R or FR	GC-MS	In condition F, phyB increases the Calvin cycle and the biosynthesis of chlorophylls, carotenoids, isoprenoid quinones, thylakoid lipids, sterols, and amino acids.	[68]
*A. thaliana* leaf	High light or low light	GC-MS	The number of plastid particles in the phyAB double mutant is reduced, and the oxidation reaction is inhibited.	[69]
*A. thaliana* seedling	Normal growth conditions	GC-MS	Phytochrome changes the diurnal growth ratio of plants.	[70]
Tomato seedling	In darkness or under continuous FR	GC-MS	PhyA is the primary regulator of tomato under FR.	[61]
*Solanum melongena* L. seed	He–Ne laser irradiation	LC-MS	He-Ne laser irradiation can break the dormant period of seeds in advance.	[71]
*A. thaliana* seedling	R or FR	LC-MS	Primary and secondary metabolites, such as niacin, alkaloids, phenylpropanoids, glucosinolates (GSLs), and flavonoids, are all affected in yellowing seedlings.	[72]
*Oryza sativa* L. seed	Normal growth conditions	UPLC-MS/MS	The absence of phyB in rice promotes the improvement in rice seed quality.	[73]

iTRAQ: isobaric tags for relative and absolute quantification; MALDI-TOF-TOF MS: matrix-assisted laser desorption/ionization–tandem time-of-flight mass spectrometry; MALDI-TOF MS: matrix-assisted laser desorption/ionization time-of-flight mass spectrometry; RPLC-MS: reverse-phase liquid chromatography–mass spectrometry; MS: mass spectrometry; LC-MS: liquid chromatography–mass spectrometry; GC-MS: gas chromatography–mass spectrometry; UPLC-MS/MS: ultra-performance liquid chromatography–tandem mass spectrometry.

## Data Availability

The data presented in this study are available upon request from the corresponding author.
